# Active contours driven by difference of Gaussians

**DOI:** 10.1038/s41598-017-14502-w

**Published:** 2017-11-03

**Authors:** Farhan Akram, Miguel Angel Garcia, Domenec Puig

**Affiliations:** 1 Department of Computer Engineering and Mathematics, Rovira i Virgili University, Tarragona, 43007 Spain; 2 Department of Electronic and Communications Technology, Autonomous University of Madrid, Madrid, 28049 Spain; 3 Bioinformatics Institute, 30 Biopolis Street, #07-01, Matrix, 138671 Singapore

## Abstract

In this paper, a novel edge-based active contour method is proposed based on the difference of Gaussians (DoG) to segment intensity inhomogeneous images. DoG is known as a feature enhancement tool, which can enhance the edges of an image. However, in the proposed energy functional it is used as an edge-indicator parameter, which acts like a balloon force during the level-set curve evolution process. In the proposed formulation, the internal energy term penalizes the deviation of the level-set function from a signed distance function and external energy term evolves the contour towards the boundaries of the objects. There are three main advantages of the proposed method. First, image difference computed using the DoG function provides the global structure of an image, which helps to segment the image globally that the traditional edge-based methods are unable to do. Second, it has a low time complexity compared to the state-of-the-art active contours developed in the context of intensity inhomogeneity. Third, it is not sensitive to the initial position of contour. Experimental results using both synthetic and real brain magnetic resonance (MR) images show that the proposed method yields better segmentation results compared to the state-of-the-art.

## Introduction

In computer vision and image processing, segmentation is a process of partitioning a digital image into multiple non-overlapping regions. The main goal is to simplify or change the image into something more meaningful or easier to analyse. Intensity inhomogeneity is one of the well-known problems in image segmentation, which can substantially reduce the accuracy of intensity based segmentation methods. It manifests as a smooth intensity variation across the image that complicates the segmentation of the objects contained in it.

To date numerous segmentation methods have been proposed for example, thresholding^1^, clustering methods^2,3^, histogram based methods^4^, edge detection^5^, region growing methods^6,7^, variational and partial differential equation (PDE) based methods (level-sets and active contours)^8–13^.

Active contours is one of the well-known aforementioned segmentation methods. It was proposed by Kass *et al*.^8^ to extract the interesting objects in an image, by evolving a level-set curve towards the object boundary. The active contours are represented as parametrized curves in a Lagrangian framework^8^ and the implicit curves in an Eulerian framework^12–15^. The main idea behind the active contours is to formulate an energy functional by using image statistics, curvature and gradient information. The energy functional is then minimized to evolve the level-set curve towards the desired object boundary. To date various active contour models and enhanced versions are employed in various image processing applications, as well as medical image analysis.

Active contour models are further divided into two main categories edge-based^8,10,15,16^ and region-based^12,17–19^ models. Both types have both strengths and limitations. Edge-based methods, as their name states use image edge information as a balloon force to evolve the curve towards the object boundaries. Level set method without re-initialization (LSWR) and distance regularized level set (DRLS) methods are devised by Li *et al*. to segment object from the image and remove the re-initialization step during the curve evolution^8,10^. However, these types of methods are unable to segment object with weak and/or blurred boundaries.

In turn, region-based methods by using image statistical information can properly segment images with weak and/or blurred boundaries. Traditional region-based methods^12,13^ are formulated with an assumption that images are homogeneous; therefore, they cannot segment intensity inhomogeneous images.

Numerous region-based methods are proposed to segment intensity inhomogeneous images by introducing the image local information in their models^20–23^. A region scalable fitting (RSF) method for image segmentation is proposed in the context of intensity inhomogeneity^20,21^. In this method, a Gaussian kernel is introduced in the energy formulation to exploit the image local information. A localized active contour method (LAC) is devised in which global region-based methods are reformulated by replacing the global means with the image local information^24^. These methods can segment intensity inhomogeneous regions, unlike their global counterparts. However, these methods are sensitive to the position of the initial contour. Moreover, they also have high computational cost due to the complicated local information in their formulation.

A local active contour model for segmenting images with intensity inhomogeneity was proposed by Zhang *et al*. in^22^. Local image information is used to define a local image fitting (LIF) energy functional, which can be interpreted as a constraint on the differences between the fitting image^20,21^ and the original image. Furthermore, a new method is used to regularize the level-set function by using Gaussian kernel filtering after each iteration. In addition, re-initialization of the level-set curve is not required. However, this method is sensitive to the initial position of contour and the final contour doesn’t strictly follow the object boundary.

Alternatively, a region-based active contour method is formulated in the context of intensity inhomogeneity by utilizing local intensity means^23^. It uses a signed pressure force (SPF) function based on a local fitted image in its energy formulation in order to segment images with intensity inhomogeneity. A Gaussian kernel is used to smooth the level-set function after every step. Therefore, this method does not require re-initialization. This method has high time complexity compared to the traditional global region-based methods^12,13^.

A variational level-set approach for bias correction and segmentation (VLSBCS) for images corrupted with intensity inhomogeneity was proposed by Li *et al*. in^17,25^. The computed bias field is intrinsically ensured to be smooth by the data term in the variational formulation, without any additional effect to maintain the smoothness of the bias field. A local statistical active contour model (LSACM) for image segmentation in the presence of intensity inhomogeneity was proposed by Zhang *et al*. in^26,27^. In this work, the inhomogeneous objects are modelled as Gaussian distributions of different means and variances. A statistical energy functional is then defined for each local region, which combines the bias field, the level-set function, and the constant approximating the true signal of the corresponding object. Both of these methods are able to segment and correct intensity inhomogeneous images. However, these are also sensitive to the initial position of contours and have high time complexity.

A hybrid region-based active contours driven by local and global fitted image models (LGFIM) was proposed in the context of intensity inhomogeneity^28^. Local and global intensity information were used to both correct and segment the inhomogeneous regions. In this paper, two SPF functions i.e., local and global were also devised to stabilize the gradient descent solution. This method is not sensitive to the initial position of the contour; therefore, it provides similar segmentation results irrespective to the initial contour position. Moreover, it is also able to properly segment intensity inhomogeneous images. However, it has a high time complexity.

**Figure 1:**
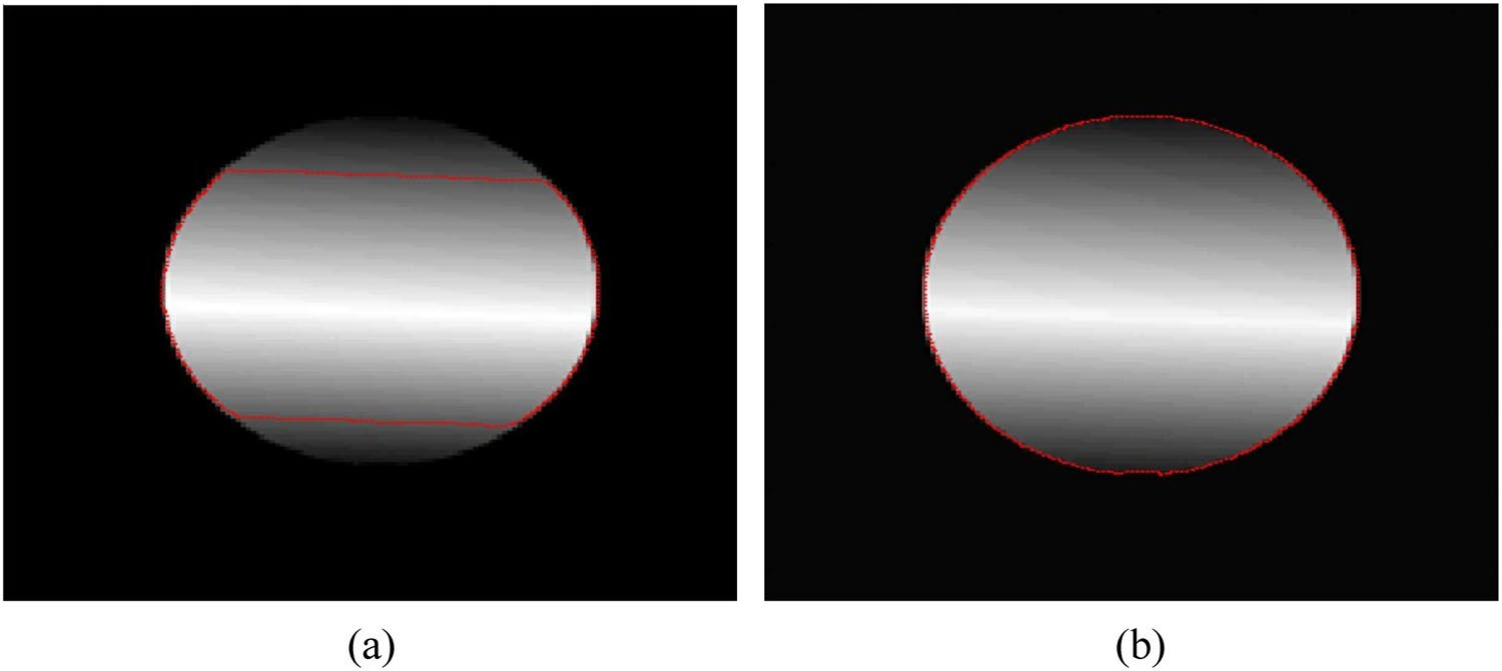
Intensity inhomogeneous image segmentation using active contours. (**a**) Image segmentation using traditional active contour method. (**b**) Image segmentation using local active contour method.

Figure 1 shows intensity inhomogeneous image segmentation using active contour method. A traditional global region-based active contour method such as, CV^12^ model is unable to segment intensity inhomogeneous object as shown in Fig. 1(a). In turn, Fig. 1(b) shows that the local region-based active contour method is able to properly segment intensity inhomogeneous object. However, local active contour methods have high time complexity because of the intensity mean computations in the local neighbourhood during the contour evolution process. Edge-based active contour methods can segment intensity inhomogeneous objects; however, these methods do not guarantee the level-set curve will stop at the object boundary, if it is blur or when the intensity difference between the background and object boundary is not clear.

In this paper, an edge-based active contour method driven by the difference of Gaussians (DoG) function is proposed in the context of intensity inhomogeneous image segmentation. The Gaussian image difference computed by the DoG function provides edge information of the global structure of the given image. This edge information is used as a balloon force in the proposed energy functional to evolve the level-set curve throughout the image structure. An energy penalizing term from^15^ is used to regularize the curve and to maintain the level-set as a signed distance function, which also removes the need for computationally expensive re-initialization of level set.

This work has three main contributions. First, it is able to segment the global structure of an image unlike traditional edge-based active contours^15,16^. Second, it is able to properly segment intensity inhomogeneous images with a low time complexity compared to local region-based methods^21–24^. Third, it is not sensitive to the initial position of the level-set curve.

The selection of the standard deviation parameter of the DoG smoothing kernels is critical, specially when the image is highly affected by noise (the parameter difference should be high). The proposed method is applied to both synthetic and real images to show the segmentation accuracy and robustness of the method.

### Proposed method

Active contours are dynamic curves that evolve toward the object boundaries to partition an image into non over-lapping regions. In traditional active contours, the curve *C* is represented by the zero level-set, such that $C=(x,y)|\varphi (x,y)=0$ of a level-set function *ϕ* (*x*, *y*). The evolution of the level-set function *ϕ* can be written in the following general form:

1 $$\frac{\partial \varphi }{\partial t}+F|\nabla \varphi |=\mathrm{0,}$$

which is referred to as the *level set equation* ^9^. Function *F* is called the force function. For image segmentation, function *F* depends on the image data and the level set function *ϕ*.

Let *I* : **Ω** → **R** ^2^ be a given image, *C* a curve at which the level-set function *ϕ*(*x*, *y*) is zero $C=(x,y)|\varphi (x,y)=0$. The energy functional *E* is defined as:

2 $$E(\varphi )={E}_{int}(\varphi )+{E}_{ext}(\varphi ),$$

In traditional edge-based active contour methods^14,29,30^, it is necessary to re-initialize (reshape) the level-set as a signed distance function during the curve evolution to properly follow and capture the object boundaries. Therefore, it is necessary to keep the evolving level-set function as an approximate signed distance function during the evolution, especially in a neighbourhood around the zero level-set. It is well-known that a signed distance function must satisfy the desirable property that $|\nabla \varphi |=1$. An energy term *P*(*ϕ*) is proposed in^15^ as a metric to characterize a function *ϕ* to a signed distance function in Ω ∈ **R** ^2^, which helps to penalize the deviation of *ϕ* from a signed distance function during its evolution. The internal energy *E*_*int*_(*ϕ*) is defined as:

3 $${E}_{int}(\varphi )=\alpha {\int }_{{\rm{\Omega }}}\frac{1}{2}{(|\nabla \varphi |-1)}^{2}dxdy,$$

where *α* is the scaling parameter of *E*_*int*_, which penalizes the energy leakage. In (2), *E*_*ext*_ is the external energy of a function *ϕ*, which is defined as follows:

4 $${E}_{ext}(\varphi )=\mu L(\varphi )+v{A}_{\Gamma }(\varphi ),$$

where *μ* > 0 and *v* are constants, and the terms *L*(*ϕ*) and *A*_Γ_(*ϕ*) are defined as:

5 $$L(\varphi )={\int }_{{\rm{\Omega }}}\,{\delta }_{\varepsilon }(\varphi )|\nabla (\varphi )|dxdy,$$

6 $${A}_{\Gamma }(\varphi )={\int }_{{\rm{\Omega }}}\,{\Gamma }_{{\sigma }_{1},{\sigma }_{2}}{H}_{\varepsilon }(-\varphi )dxdy,$$

where *H*_*ε*_(*ϕ*) is the regularized version of the Heaviside function:

7 $${H}_{\varepsilon }(\varphi )=\frac{1}{2}+\frac{1}{\pi }arctan(\frac{\varphi }{\varepsilon })$$

Parameter *ε* controls the smoothness of the Heaviside function. For *ε* → 0, the Heaviside function is the ideal unit step function. In (6), ${{\rm{\Gamma }}}_{{\sigma }_{1},{\sigma }_{2}}$ is the difference of Gaussian function, which is used to replace a traditional edge indicator function. In (5), *δ*_*ε*_(*ϕ*) is the smooth version of the Dirac function, defined as:

8 $${\delta }_{\varepsilon }(\varphi )=\frac{\varepsilon }{\pi ({\varphi }^{2}+{\varepsilon }^{2})}$$

where parameter *ε* controls the width of the Dirac function. For *ε* → 0, the Dirac function is the ideal unit impulse.

In this paper, a level-set method based on the difference of Gaussians (DoG) is proposed. A DoG function, which is equivalent to the Mexican Hat function, is a feature enhancement tool that involves the subtraction of a blurred version of an original image from another, less blurred version of the original. As a feature enhancement algorithm, the difference of Gaussian functions can be utilized to increase the visibility of edges and other details present in a digital image. The difference of Gaussians algorithm removes high frequency detail that often includes random noise, thus rendering this approach one of the most suitable for processing images with a high degree of noise. A major drawback of the application of the algorithm is an inherent reduction of the overall image contrast that results. In this work, it is employed as an edge-detector, which works as a balloon force in the external term of the proposed energy formulation during the level-set curve evolution. Let *I* : Ω → **R** ^2^ be an input image. The DoG function ${\Gamma }_{{\sigma }_{1},{\sigma }_{2}}(x)$ is then defined as:

9 $$\begin{array}{rcl}{\Gamma }_{{\sigma }_{1},{\sigma }_{2}}(x) & = & I(x)\ast \frac{1}{{\sigma }_{1}\sqrt{2\pi \,}}\exp \,(-\frac{{x}^{2}}{2{\sigma }_{1}^{2}})\\  &  & -I(x)\ast \frac{1}{{\sigma }_{2}\sqrt{2\pi \,}}\exp \,(-\frac{{x}^{2}}{2{\sigma }_{2}^{2}}),\end{array}$$

where *σ*_1_ and *σ*_2_ are the standard deviations of the first and second Gaussian kernels, respectively, where *σ*_1_ < *σ*_2_.

**Figure 2:**
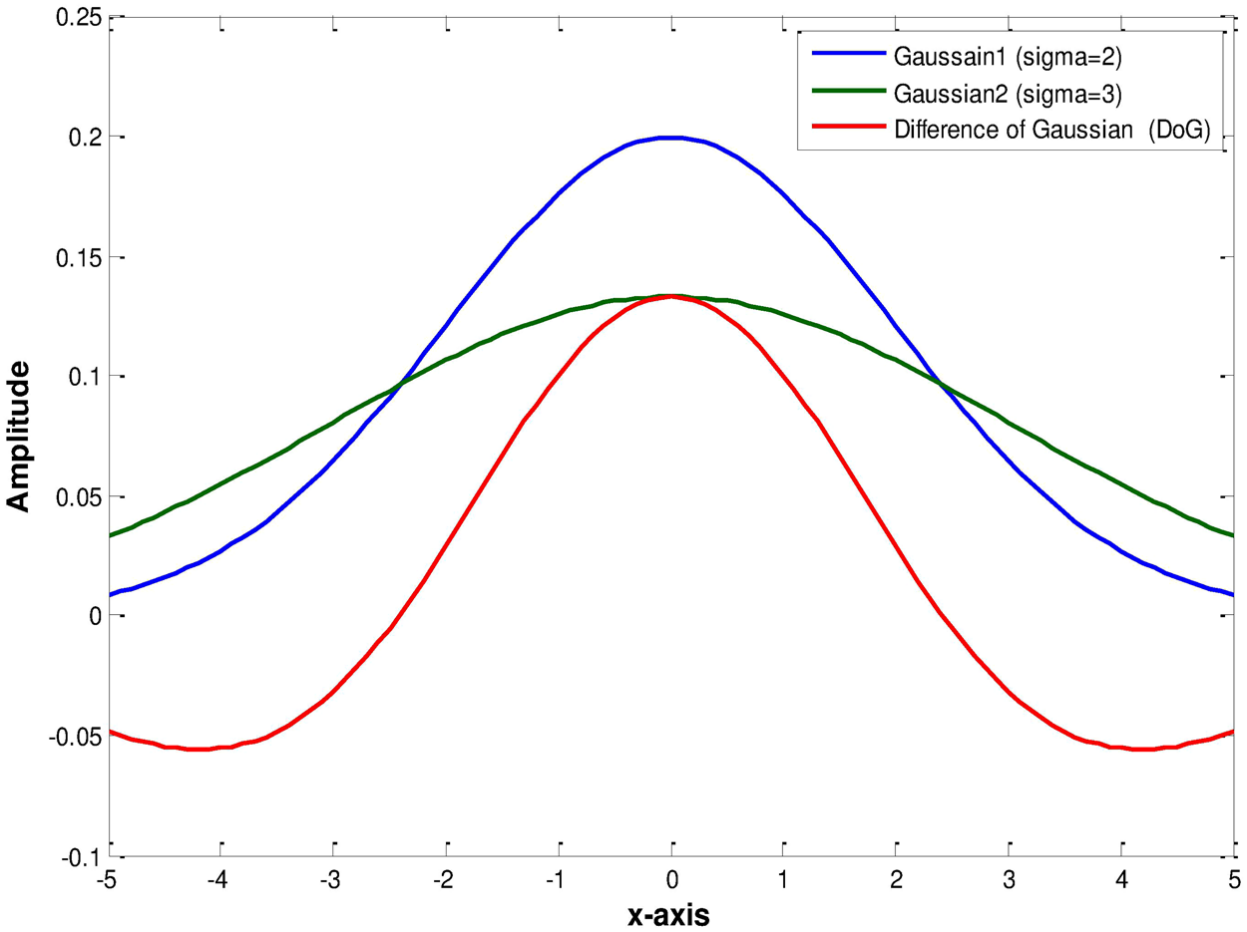
1D difference of Gaussians (DoG) function.

Figure 2 shows a 1D difference of Gaussians (DoG) function. It shows that the DoG function is zero when the slopes of both Gaussian functions intersect with each other. It helps to extract edges even when the images contain intensity inhomogeneous objects.

Finally, from (2), the proposed energy functional, which uses the difference of Gaussians (DoG) function ${\Gamma }_{{\sigma }_{1},{\sigma }_{2}}(x)$ to extract edge information, is defined as:

10 $$\begin{array}{rcl}E(\varphi ) & = & v\,{\int }_{{\rm{\Omega }}}\,{{\rm{\Gamma }}}_{{\sigma }_{1},{\sigma }_{2}}(x){H}_{\varepsilon }(-\varphi )dx+\mu \,{\int }_{{\rm{\Omega }}}\,|\nabla {H}_{\varepsilon }(\varphi )|dx\\  &  & +\alpha \,{\int }_{{\rm{\Omega }}}\,\frac{1}{2}{(|\nabla \varphi |-1)}^{2}dx,\end{array}$$

In the above equation, the first term detects the edges using the DoG function. The second term regularizes the region and the third term penalizes the energy leakage. The first variation of the Gateaux derivative^31^ of the functional *E* is denoted by $\frac{\partial E}{\partial \varphi }$, which has the following relationship with the evolution equation:

11 $$\frac{\partial \varphi }{\partial t}=-\frac{\partial E}{\partial \varphi },$$

The above equation is the gradient descent flow that minimizes the energy functional *E*. For a particular functional *E*(*ϕ*) explicitly defined in terms of *ϕ*, the Gateaux derivative can be computed and expressed in terms of function *ϕ* and its derivatives^31^.

By calculus of variations^31^, the Gateaux derivative (first variation) of the functional *E* in (10) can be written as:

12 $$\frac{\partial E}{\partial \varphi }=-(v{{\rm{\Gamma }}}_{{\sigma }_{1},{\sigma }_{2}}+\mu \,{\rm{div}}(\frac{\nabla \varphi }{|\nabla \varphi |})){\delta }_{\varepsilon }(\varphi )+\alpha [{\rm{\Delta }}\varphi -{\rm{div}}(\frac{\nabla \varphi }{|\nabla \varphi |})],$$

where ∇ is the Laplacian operator. Therefore, the function *ϕ* that minimizes this functional satisfies the Euler-Lagrange equation $-\frac{\partial E}{\partial \varphi }=0$. The steepest descent process for minimization of the functional *E* yields the following gradient flow:

13 $$\frac{\partial \varphi }{\partial t}=(v{\Gamma }_{{\sigma }_{1},{\sigma }_{2}}+\mu \,{\rm{div}}(\frac{\nabla \varphi }{|\nabla \varphi |})){\delta }_{\varepsilon }(\varphi )+\alpha [{\rm{\Delta }}\varphi -{\rm{div}}(\frac{\nabla \varphi }{|\nabla \varphi |})],$$

In this paper, the spatial partial derivatives $\frac{\partial \varphi }{\partial x}$ and $\frac{\partial \varphi }{\partial y}$ are approximated by the central difference. The approximation of (13) using a central difference scheme can be written as:

14 $$\frac{{\varphi }_{i,j}^{k+1}-{\varphi }_{i,j}^{k}}{\tau }=\xi ({\varphi }_{i,j}^{k})$$

where $\xi ({\varphi }_{i,j}^{k})$ is the approximation of the right hand side in (13) by the above difference scheme. The difference equation in (14) can be expressed as the following iteration:

15 $${\varphi }_{i,j}^{k+1}={\varphi }_{i,j}^{k}+\tau \xi ({\varphi }_{i,j}^{k})$$

where *τ* is the time step used in the above numerical implementations. There is a close relation between the time step and the scaling parameter of the energy penalization term i.e., *τ* × *α* ≤ 0.2.

In level-set methods, it is essential to initialize the level-set function *ϕ* as a signed distance function (SDF) *ϕ*_0_. In the proposed formulation, not only is the re-initialization procedure completely eliminated, but the level-set function *ϕ* no longer needs to be initialized as an SDF. The initial level-set function *ϕ*_0_ is defined as:

16 $$\varphi (x,t\mathrm{=0})\,=\,\{\begin{array}{ll}-\rho , & x\in {{\rm{\Omega }}}_{0}-\partial {{\rm{\Omega }}}_{0}\\ \mathrm{0,} & x\in \partial {{\rm{\Omega }}}_{0}\\ \rho , & x\in {\rm{\Omega }}-{{\rm{\Omega }}}_{0}\end{array}$$

**Figure 3:**
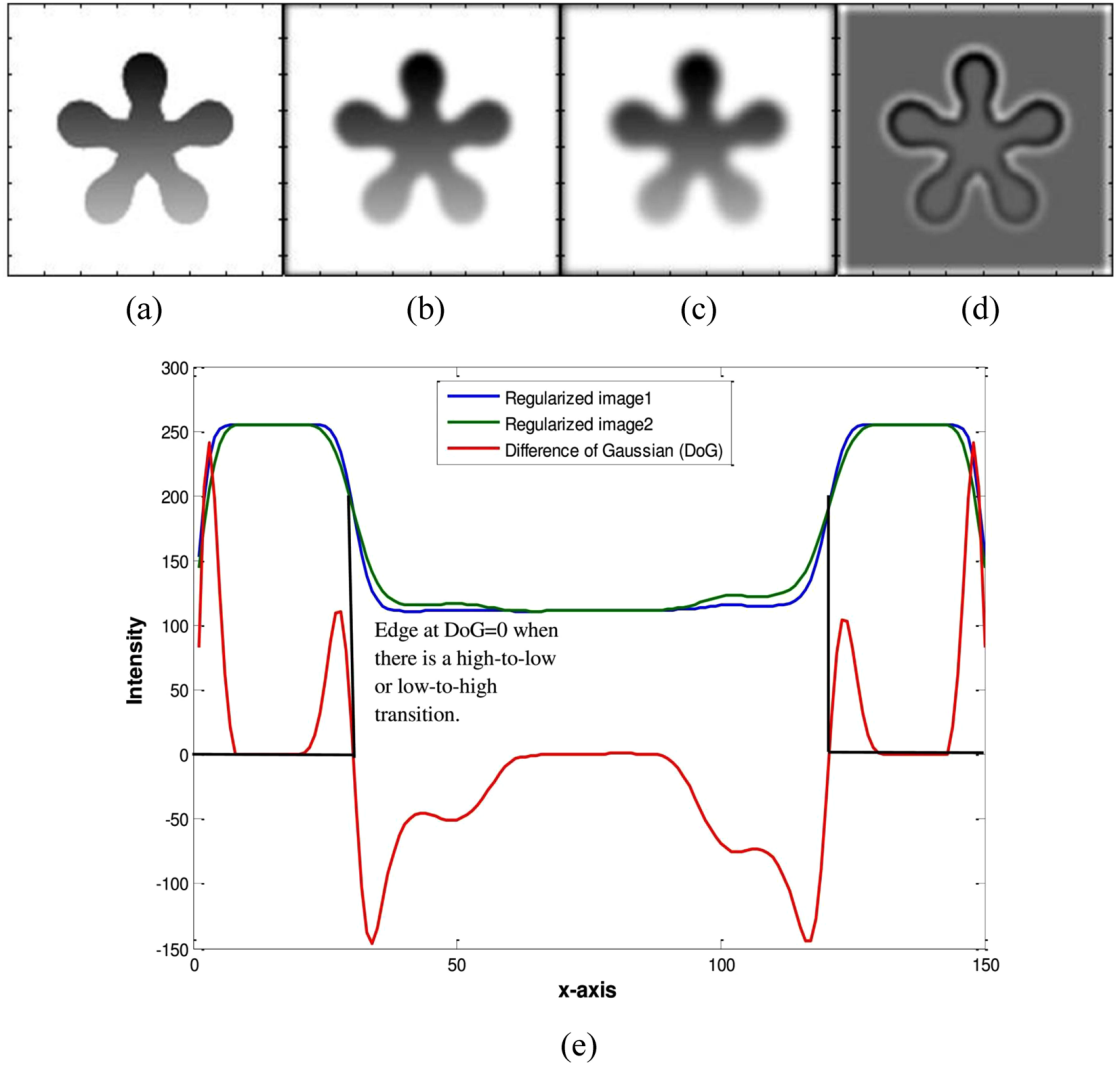
DoG of the middle slice of the flower image. (**a**) Original image, (**b**) Gaussian regularized image (*σ* = 2), (**c**) Gaussian regularized image (*σ* = 3), (**d**) Difference of Gaussian regularized images (DoG), (**e**) middle profile comparison of (**b**–**d**).

In (16), *ρ* > 0 is a constant (*ρ* = 1 in this work) and *t* = 0 define the initial condition of the level-set function *ϕ*_0_ = *ϕ*(*x*,*t* = 0). Ω_0_ is the inner region of the initial level set *ϕ*_0_, Ω is the image domain and ∂Ω_0_ the boundary of level set *ϕ*. Figure 3 shows a 1D profile from the middle column of the difference of Gaussian regularized image shown in Fig. 3(d). Figure 3(b) and (c) are Gaussian regularized images, which are subtracted to produce the DoG based edge profile of the given image, as shown in Fig. 3(d). Figure 3(e) shows a middle profile comparison between Fig. 3(b)–(d). It shows that when there is an intersection between the 1D middle profile of Fig. 3(b) and (c), then the edge is detected (a transition from high to low or vice versa), which is shown with the red line in Fig. 3(e).

## Results

In this section, segmentation results using both synthetic and real images are discussed. The proposed method is implemented using MATLAB and run on a 3.4 GHz Intel Core-i7 with 16 GB of RAM, testing it on both synthetic images and real brain magnetic resonance (MR) images of 250 × 250 pixels with 256 grey levels (8bpp). The parameters used in all experiments in this section are: *μ* = 0.001 × 255^2^, *v* = −40, *σ*_1_ = 1, *σ*_2_ = 2, *α* = 1.0, *ε* = 1 and the time step *τ* = 0.1.

**Figure 4:**
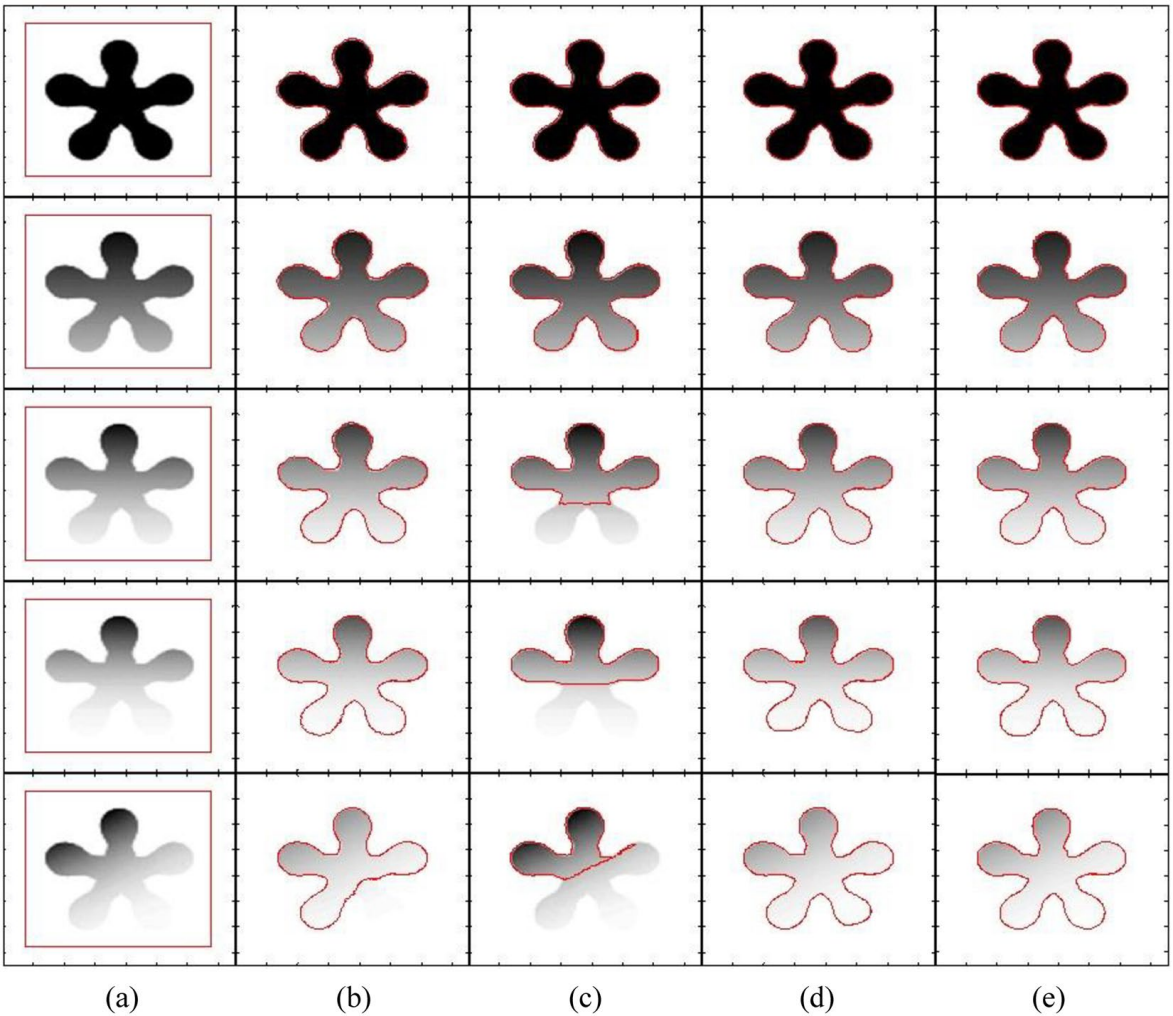
Segmentation of a flower object using different contrast variations (from homogeneous to inhomogeneous). (**a**) Initial contour, (**b**) DRLS, (**c**) CV, (**d**) RSF, (**e**) Proposed method.

Figure 4 shows segmentation results of different state-of-the-art methods using a flower image with different contrast variations until the flower object becomes inhomogeneous with respect to the image background. It shows that the DRLS method is able to properly segment images in the first four rows, but it is unable to segment the image in the last column. In turn, the CV method is able to properly segment the images in the first two rows and fails to properly segment the remaining images. Although the RSF method is able to segment objects in all images, the petals of the flowers in the images of the last two rows are not properly segmented. In turn, the qualitative comparison shows that the proposed method yields the best segmentation result for all the objects.

Table 1 shows a CPU time comparison among the evaluated methods tested in Fig. 4. For the images in the first and second rows, the CV method is the fastest among all segmentation algorithms. In turn, the proposed method is the fastest for the images in the last three rows.

**Table 1:** CPU time comparison among the state-of-the-art methods shown in Fig. 4.

Row number	DRLS		CV		RSF		Proposed	
	Iterations	CPU time (s)	Iterations	CPU time (s)	Iterations	CPU time (s)	Iterations	CPU time (s)
1	500	5.45	20	**0.80**	200	6.81	140	1.44
2	500	4.79	20	**0.81**	250	8.45	140	1.38
3	500	4.83	1500	12.61	300	10.38	140	**1.40**
4	1300	13.66	1200	10.09	300	9.81	140	**1.39**
5	900	8.77	2500	20.30	550	17.38	200	**1.97**

**Figure 5:**
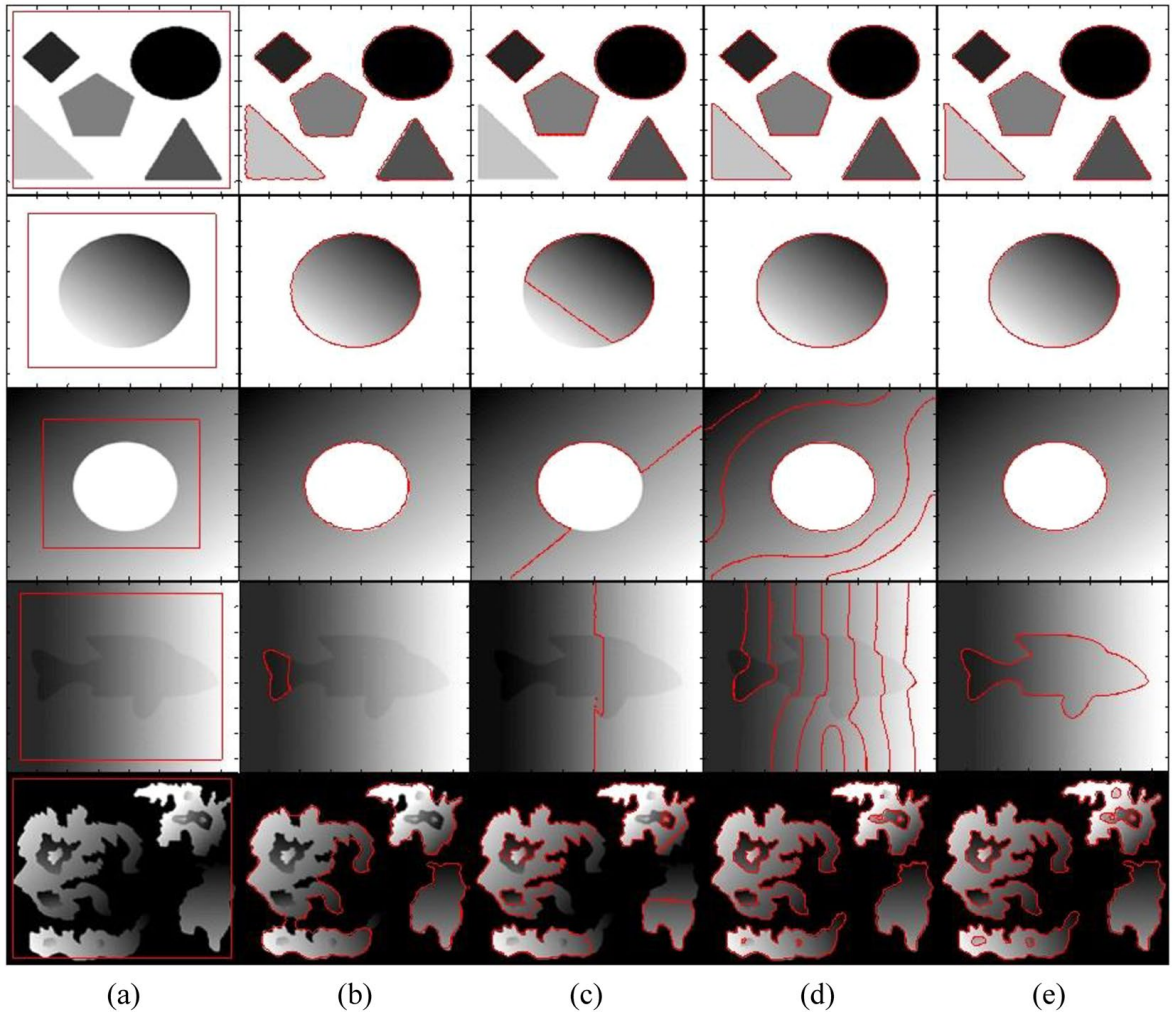
Image segmentation with different types of intensity inhomogeneity (from homogeneous to inhomogeneous). (**a**) Initial contour, (**b**) DRLS, (**c**) CV, (d) RSF, (**e**) Proposed method.

Figure 5 shows that the DLSR method could segment the first three images properly. The CV method could not properly segment the objects in all of the images. The RSF method could segment the first two images and the last properly. However, in the last image, the final contour has missed some details of the small objects enclosed in the main object. Moreover, some details of the big object in the right corner are also missed. In turn, the proposed method yielded the best segmentation results for all of the images.

Table 2 shows a CPU time comparison among the evaluated methods tested in Fig. 5. It shows that the CV method is the fastest among all segmentation algorithms for the images in the first and the last rows. For the second and fourth images, the proposed method is the fastest. In turn, for the image in the third row, the DRLS method is the fastest.

**Table 2:** CPU time comparison among the state-of-the-art methods shown in Fig. 5.

Row number	DRLS		CV		RSF		Proposed	
	Iterations	CPU time (s)	Iterations	CPU time (s)	Iterations	CPU time (s)	Iterations	CPU time (s)
1	400	4.83	20	**0.88**	100	3.81	240	2.87
2	200	2.28	500	4.77	200	3.59	80	**1.08**
3	200	**2.16**	500	4.97	300	6.31	400	3.73
4	1100	13.3	500	4.83	500	10.64	350	**3.59**
5	1200	22.78	500	**5.42**	300	6.44	350	5.73

**Figure 6:**
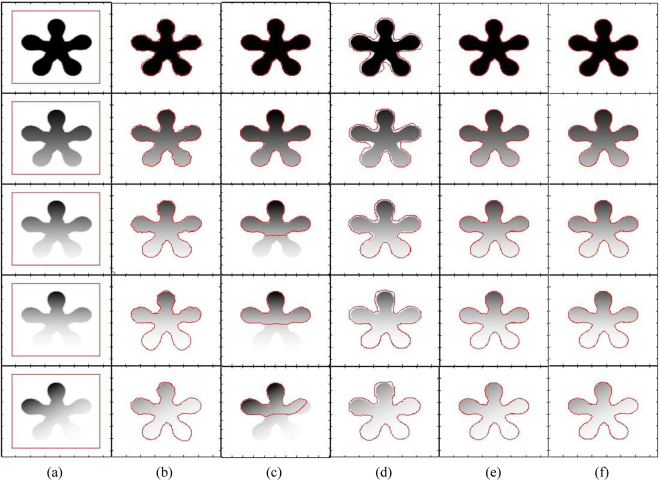
Image segmentation with different types of intensity inhomogeneity (from homogeneous to inhomogeneous). (**a**) Initial contour, (**b**) LIF, (**c**) VLSBCS, (**d**) LSACM, (**e**) LGFIM (**f**) Proposed method.

Figure 6 shows that only the proposed method and LGFIM are able to accurately segment all of the intensity inhomogeneous objects. LIF and LSACM are also able to segment all the objects. However, for both methods, the level-set curve around the boundaries of the objects is not quite smooth, which results in information loss. In turn, the VLSBCS method is able to properly segment the images in the first two rows and fails in the last three rows.

Table 3 shows a CPU time comparison between the evaluated local active contour methods shown in Fig. 6. It shows for all images the proposed method yields the lowest CPU time; therefore, it is the fastest.

**Table 3:** .CPU time comparison with the local active contour methods shown in Fig. 6.

Row number	LIF		VLSBCS		LSACM		LGFIM		Proposed	
	Iterations	CPU time (s)	Iterations	CPU time (s)	Iterations	CPU time (s)	Iterations	CPU time (s)	Iterations	CPU time (s)
1	400	5.45	20	1.71	40	24.69	20	1.58	140	**1.44**
2	450	5.97	30	2.21	40	25.55	30	2.19	140	**1.38**
3	600	7.56	30	2.27	50	32.05	70	4.12	140	**1.40**
4	2000	57.38	100	5.87	60	38.69	90	5.09	140	**1.39**
5	2000	55.91	100	6.31	80	50.72	100	5.78	200	**1.97**

**Figure 7:**
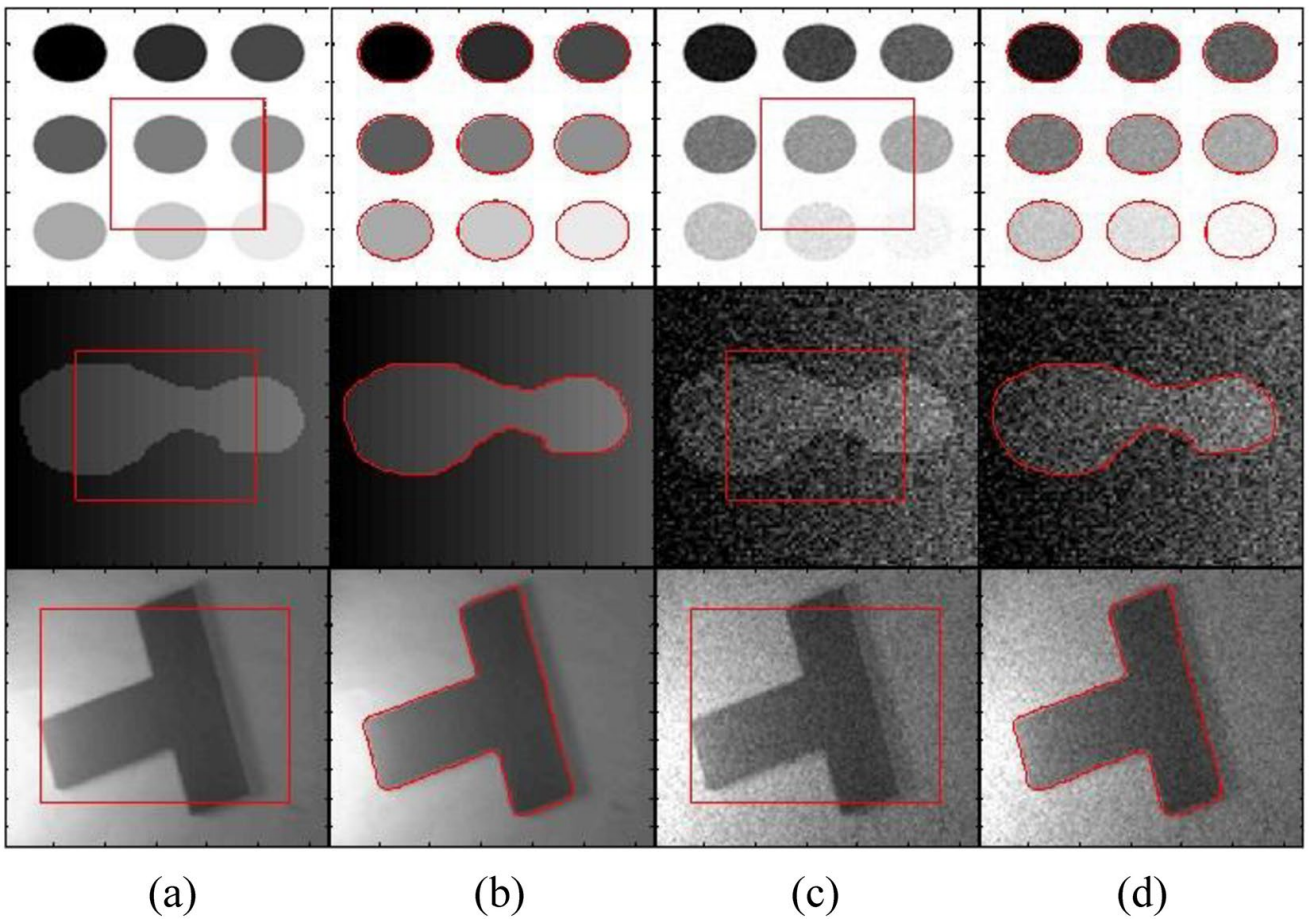
Segmentation results of real and noisy images using the proposed method. (**a**) and (**c**) Initial contour, (**b**) and (**d**) Final contour.

In Fig. 7, two images with and without noise are used to show the segmentation capability of the proposed method in the presence of noise. Images shown in the first and third rows are affected by the Poisson noise. Whereas, image in the second row is affected by the Gaussian noise with mean = 0.01 and variance = 0.2. The propose method is able to properly segment images with Poisson noise by using the default values of *σ*_1_ and *σ*_2_ as discussed earlier in this section. However, in case of Gaussian noise different values of *σ*_1_ and *σ*_2_ are used i.e., *σ*_1_ = 2, *σ*_2_ = 10. In order to segment images with intense noise, bigger values of *σ*_1_ and *σ*_2_ are used. Moreover, regularization parameter *μ* is also increased (*μ* = 0.01 × 255 × 255). It concludes that the proposed method is not affected by the presence of noise. It is able to properly segment the object both with and without background noise.

**Figure 8:**
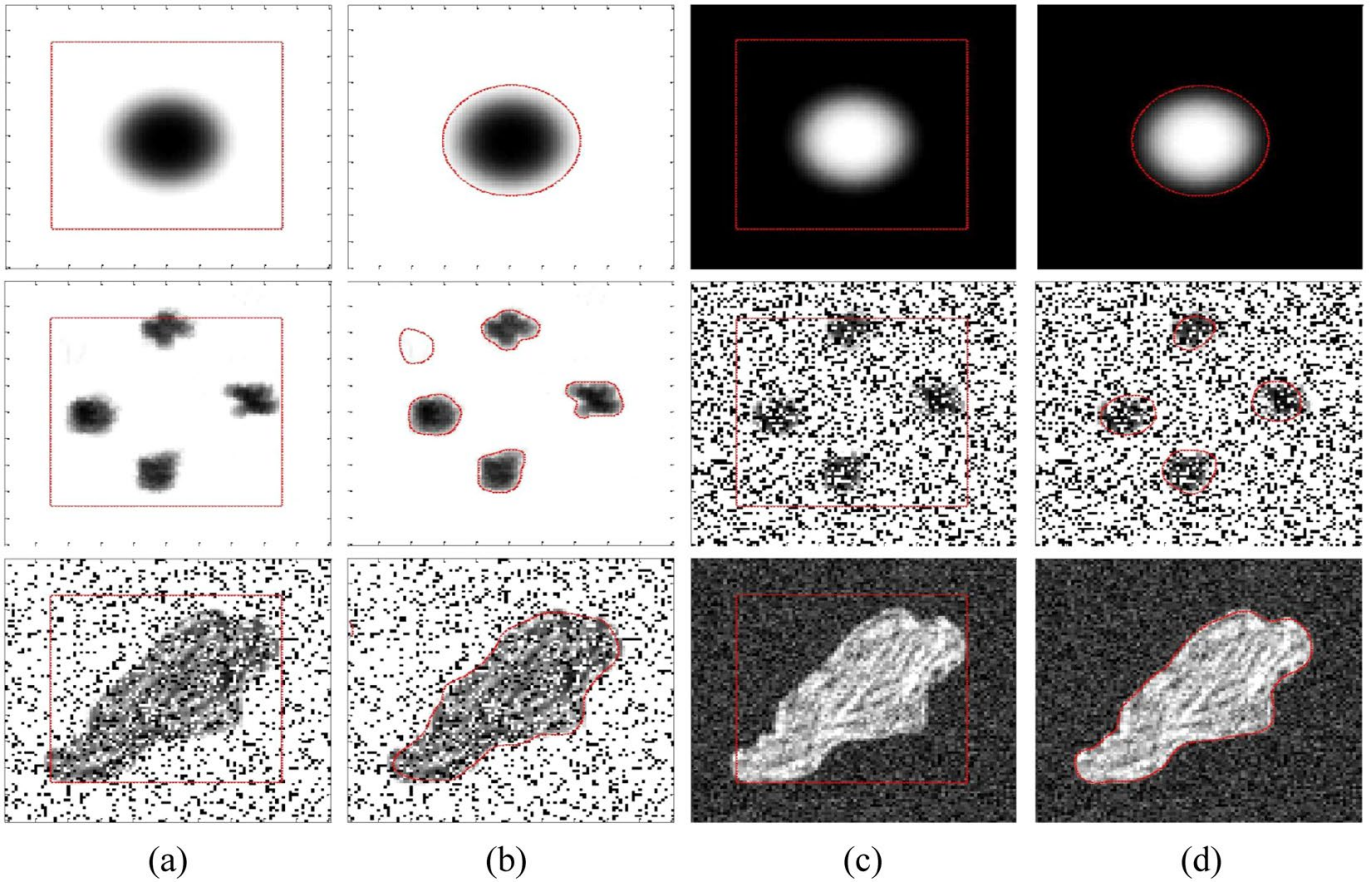
Segmentation results of images with weak boundaries, with salt and pepper and Gaussian Noise. (**a**) and (**c**) Initial contour, (**b**) and (**d**) Final contour.

Figure 8 shows different images with weak boundaries, salt and pepper and Gaussian noise. First row shows circular object with weak boundaries in both white and black background. It shows that in both cases, the proposed method is able to properly segment objects with weak boundaries. Second row shows image with five different objects with and without salt and pepper noise of *d* = 0.3. Where *d* is the noise density. Proposed method is able to properly segment all five objects with weak boundaries, when there is no noise in the image. In second case, when salt and pepper noise of *d* = 0.3 is applied; the proposed method could only segment four objects out of five. The fifth object was dissipated by the noise. In last row, both salt and pepper and Gaussian noise are applied to an irregular object with weak boundaries. In the first image, a salt and pepper noise of *d* = 0.2 is applied. In the second image, Gaussian noise of zero mean and variance = 0.3 is applied. The last row shows that the proposed method yields acceptable segmentation results for both cases of noise.

**Figure 9:**
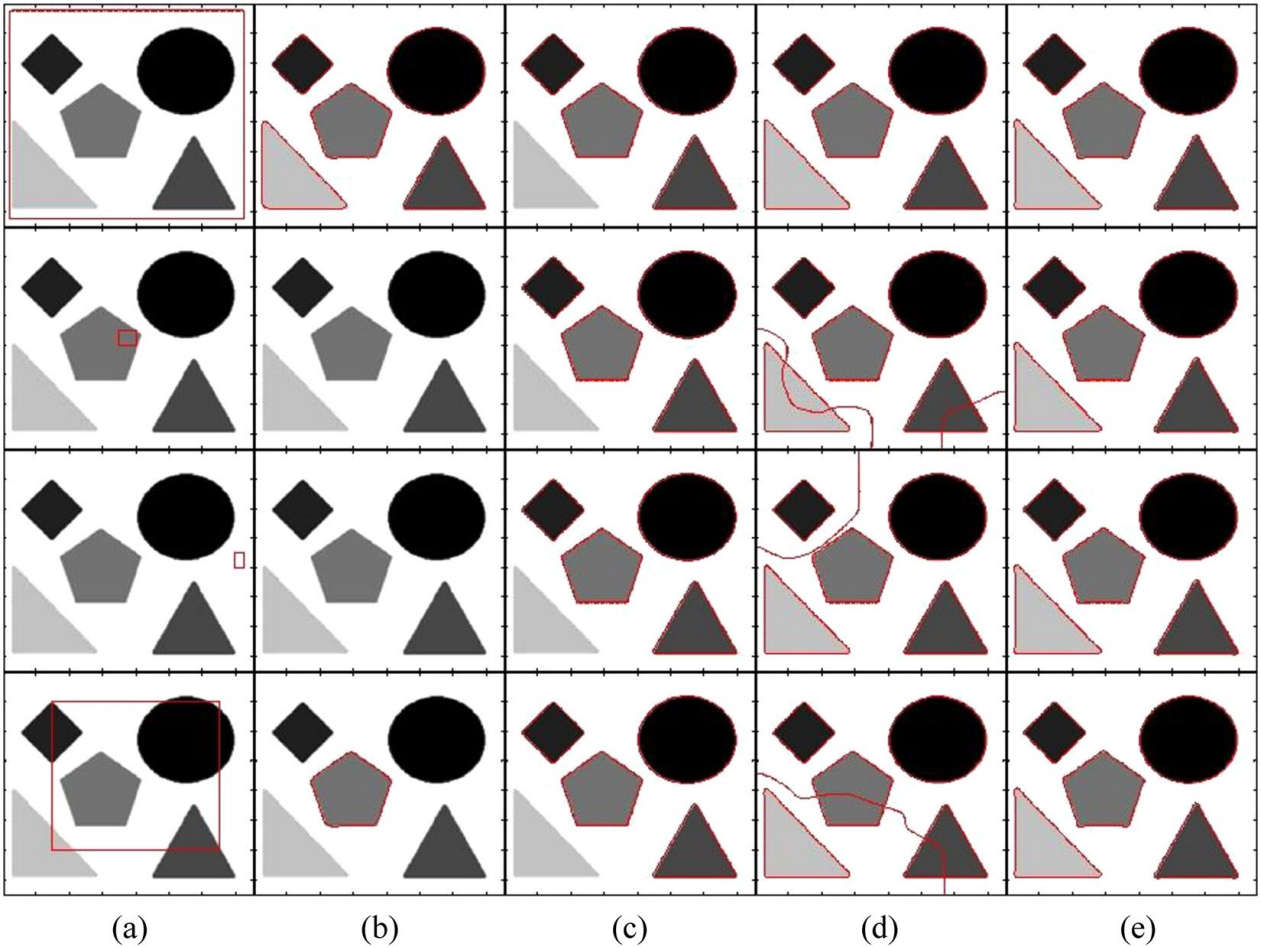
Effects of initial contour on final segmentation. (**a**) Initial contour, (**b**) DRLS, (**c**) CV, (d) RSF, (**e**) Proposed method.

Figure 9 shows the impact of the position of the initial contour on the evaluated methods. The segmentation results produced by the proposed method are not affected by the initial position of the contour, unlike the other evaluated methods, which are sensitive to the initial contour.

**Figure 10:**
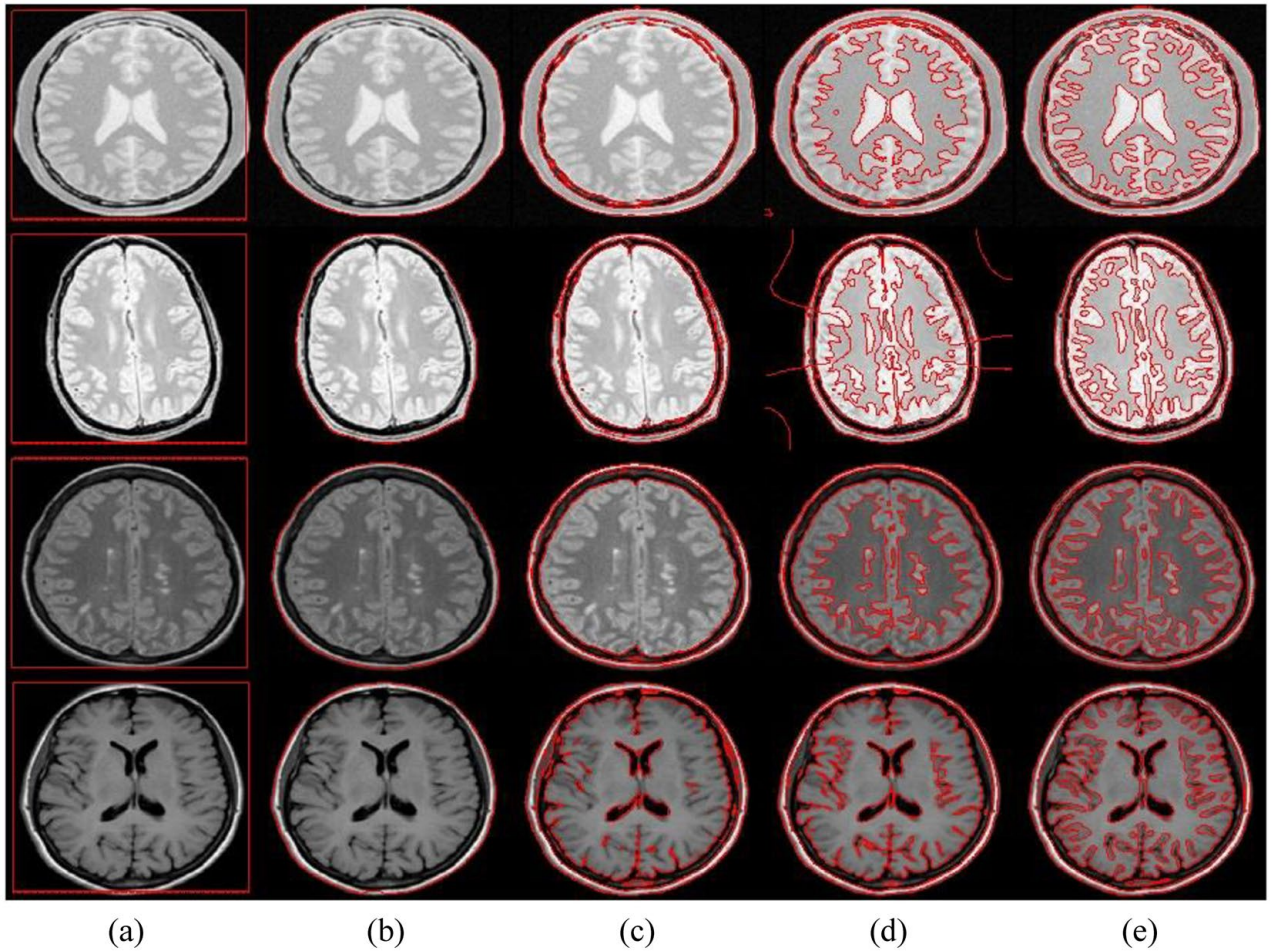
Brain MR image segmentation comparison with the state-of-the-art. (**a**) Initial contour, (**b**) DRLS, (**c**) CV, (**d**) RSF, (**e**) Proposed method.

Figure 10 shows the brain MR image segmentation problem using the evaluated methods. RSF and the proposed method are able to segment the detailed anatomical structure of the brain region, whereas the remaining methods fail to do so. There is region overlap in the segmentation result of RSF method shown in the second row, which concludes the proposed method yields the best segmentation.

### Quantitative analysis

In this section, the proposed method is quantitatively compared with the alternative state-of-the-art methods. In this paper, several metrics are used to evaluate the binary segmentation of a structure in an image. Let *G* be the ground truth of the region of interest and *S* the segmented region in the given image *I*:Ω → ***R*** ^2^. The true positive (*TP*) set is defined as $TP=G\cap S$, which is the set of the segmented region common in both *G* and *S*. The true negative (*TN*) set is defined as $TN=\bar{G}\cap \bar{S}$, which is the set of image background common in both *G* and *S*. Similarly, the false positive (*FP*) set is defined as $FP=\bar{G}\cap S$, which is the false object segmented as region of interest not belonging to set *G*. In turn, the false negative (*FN*) is defined as $FN=G\cap \bar{S}$, which is the region of interest missed by the proposed method during the segmentation process.

From the above subsets, different similarity metrics are computed. In particular, the Jaccard index (JI)^32^, the Dice coefficient (DSC)^33^ and the Matthews correlation coefficient (MCC)^34^ are frequently used in set comparison that is, to compute the segmentation accuracy when the ground truth of the region of interest is available. In this paper, the three set similarity metrics (JI, DSC and MCC) are computed for the quantitative analysis. They are defined as:

$$JI=\frac{TP}{TP+FP+FN},\,DSC=\frac{TP}{\frac{1}{2}(TP+FN+TP+FP)},$$

17 $$MCC=\frac{(TP\times TN)-(FP\times FN)}{\sqrt{(TP+FP)(TP+FN)(TN+FP)(TN+FN)}}$$

For the maximum segmentation accuracy, the values of JI, DSC and MCC should be close to 1 (ideally 1). The Hausdorff distance (HD)^35^ is another similarity metric, which is used to compute the accuracy between two sets. It provides a symmetric distance measure of the maximal discrepancy between two labelled contours and is defined as:

18 $$HD(G,S)=\,{\rm{\max }}(\mathop{{\rm{\max }}}\limits_{g\in G}(\mathop{{\rm{\min }}}\limits_{s\in S}\,d(g,s)),\mathop{{\rm{\max }}}\limits_{s\in S}(\mathop{{\rm{\min }}}\limits_{g\in G}\,d(g,s)))$$

where *G* and *S* are the ground truth and computed contours, respectively, and *d*(*g*,*s*) denotes the Euclidean distance. For the maximum segmentation accuracy, the HD value should be close to 0 (ideally 0).

**Figure 11:**
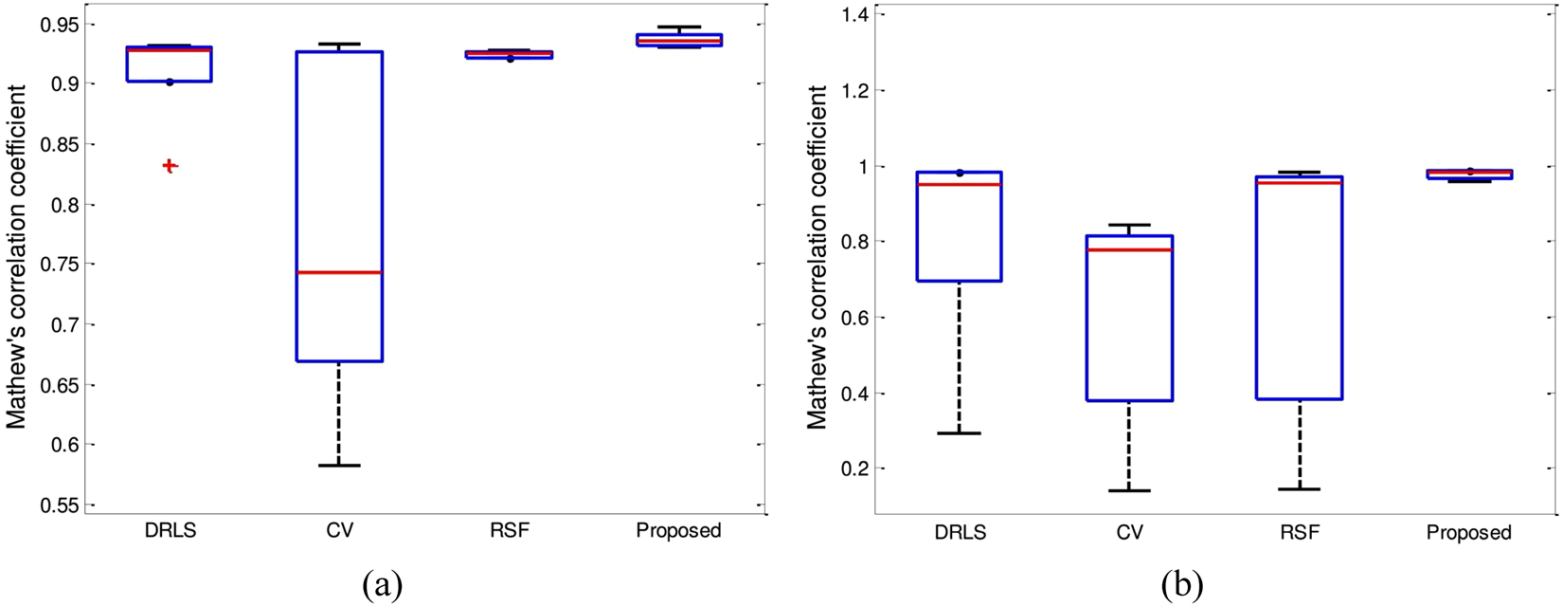
Accuracy plot for Figs 4 and 5 using the Matthews correlation coefficient. (**a**) Box plot of Fig. 4. (**b**) Box plot of Fig. 5.

Figure 11 shows the segmentation accuracy comparison using the Matthews correlation coefficient (MCC) from Figs 4 and 5 in a box plot. The proposed method yields the best segmentation results for both Figs 4 and 5. However, in Fig. 4, RSF yields a similar result compared to the proposed method.

**Figure 12:**
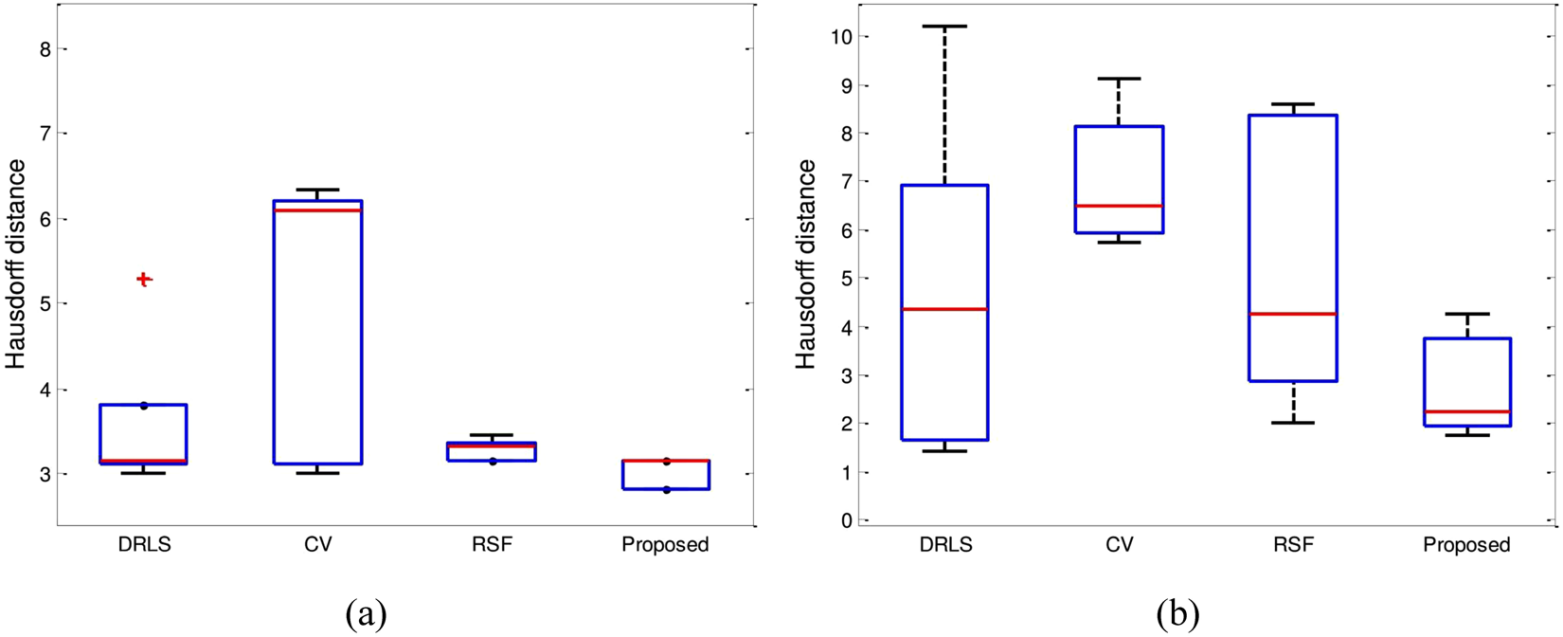
Accuracy plot for Figs 4 and 5 using the Haursdorff distance. (**a**) Box plot of Fig. 4. (**b**) Box plot of Fig. 5.

Figure 12 shows the segmentation accuracy comparison using the Hausdorff distance (HD) from Figs 4 and 5 in a box plot. It shows that the proposed method yields the smallest HD value for both Figs 4 and 5. Therefore, it yields the best segmentation results.

Table 4 shows the segmentation accuracy comparison of the proposed method with the state-of-the-art using the JI and DSC similarity metrics. Both the mean and standard deviation (mean error) of the evaluated metrics are considered in the result compilation. For Fig. 4, both the proposed method and RSF yield similar values for both JI and DSC. In turn, the proposed method yields best segmentation result for the Fig. 5.

**Table 4:** Segmentation accuracy analysis comparison using Jaccard index and Dice coefficient similarity metrics.

Figure	Jaccard index (JI)				Dice coefficient (DSC)			
	DRLS	CV	RSF	Proposed	DRLS	CV	RSF	Proposed
4	0.965 ± 0.007	0.918 ± 0.023	0.97 ± 0.001	**0.974** **±** **0.002**	0.982 ± 0.004	0.957 ± 0.012	0.985 ± 0	**0.987** **±** **0.001**
5	0.775 ± 0.169	0.612 ± 0.145	0.655 ± 0.172	**0.943** **±** **0.016**	0.814 ± 0.157	0.715 ± 0.125	0.731 ± 0.147	**0.971** **±** **0.009**

This section also shows segmentation results using 2D brain MR images from a public database of 20 brain anatomical models^36,37^. All images have 250 × 250 pixels and 8 bits per pixel. As a practical application, brain MR images are segmented into white matter (WM) and gray matter (GM) regions, which can be helpful to psychologists to pinpoint psychological diseases and to surgeons during brain surgery.

In order to partition a brain MR image into WM and GM regions, the segmentation result is split into two regions based on two phases: *ϕ* > 0 and *ϕ* < 0. The WM and GM regions represent the brain region, which is the region of interest, while the regions outside the brain (e.g., skull, fat and vacuum) can be taken as irrelevant regions. Therefore, we manually extracted the brain area to segment the WM and GM regions, removing the other irrelevant regions out of second row, the third and fourth images show the ground truths of the WM and GM regions, respectively.

**Figure 13:**
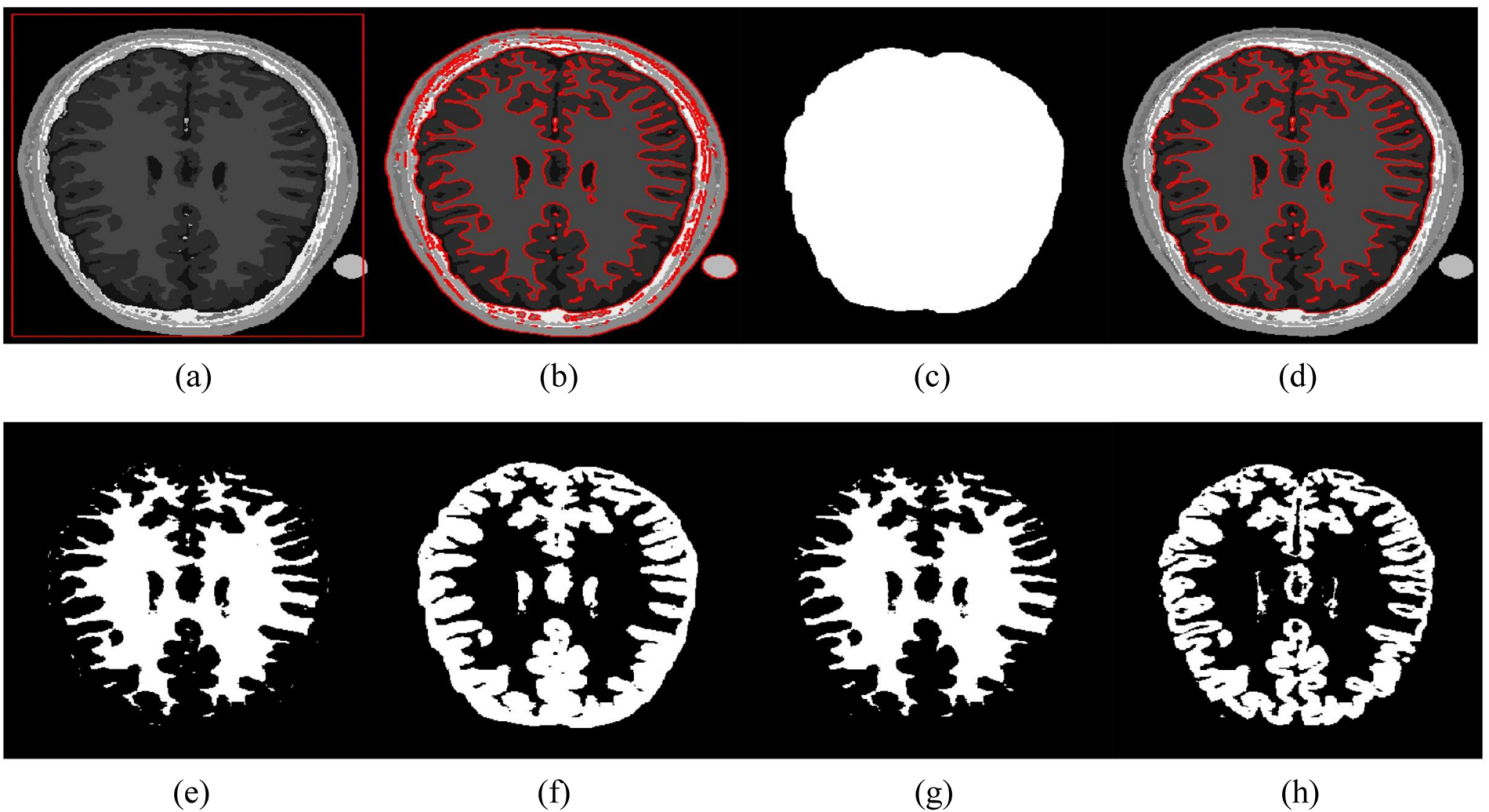
WM and GM regions computed with the proposed method and their respective ground truths. (**a**) Initial contour, (**b**) Final contour, (**c**) Brain mask, (**d**) Masked contour, (**e**) Computed WM, (**f**) Computed GM, (**g**) WM ground truth, and (**h**) GM ground truth.

**Figure 14:**
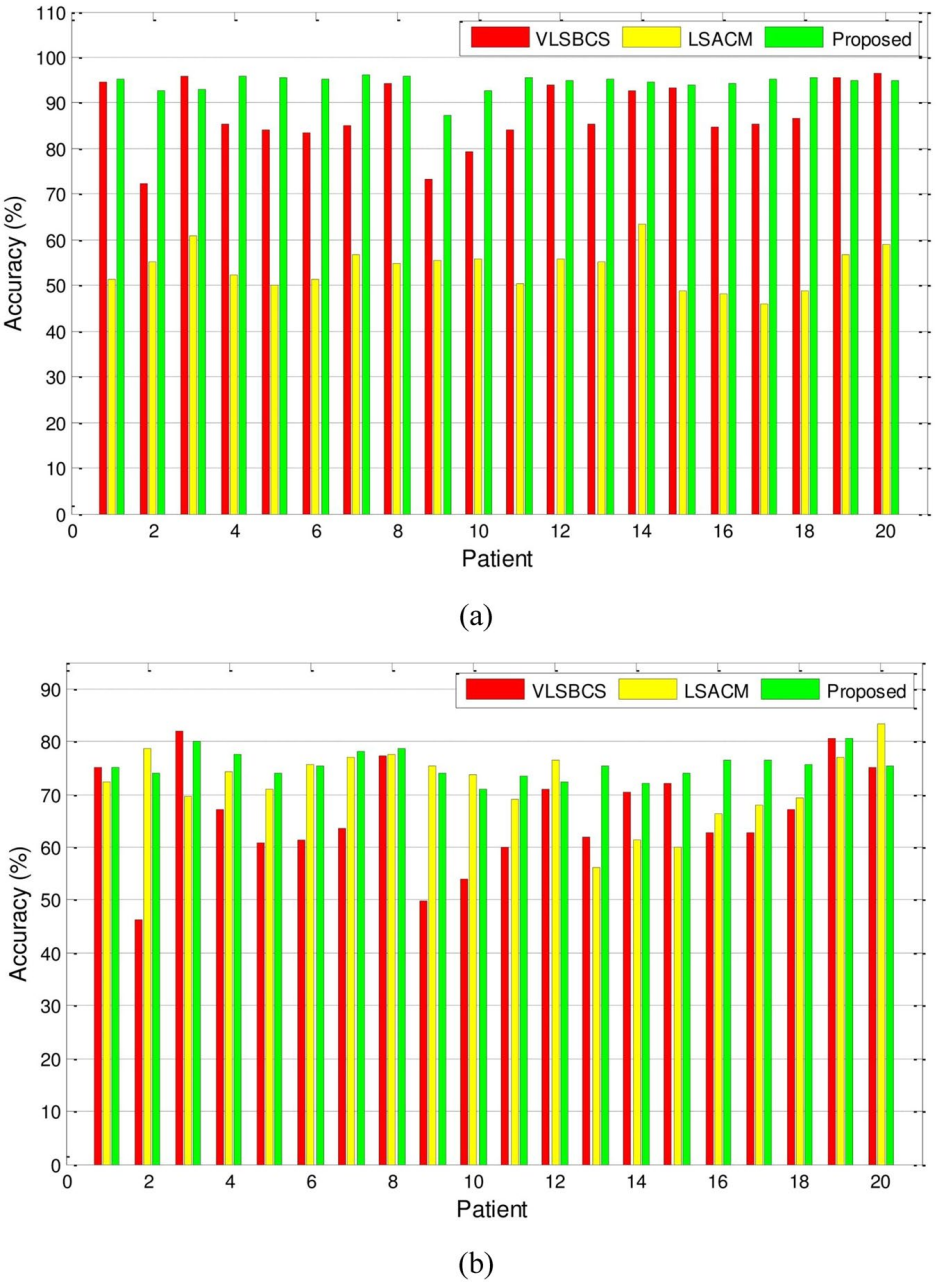
Segmentation accuracy (in terms of Jaccard index × 100) analysis of (**a**) WM, and (**b**) GM regions using two-phase active contours.

Figure 13 shows the accuracy analysis of the region of interest in the brain MR images. A total of 100 2D slices from 20 brain anatomical models^37^ were used. Five 2D slices from every patient were considered. The WM and GM regions for all methods were computed as depicted in Fig. 13. The segmentation accuracy corresponding to the WM and GM regions presented in Fig. 14 was obtained using percentage accuracy in terms of Jaccard index from (17).

Figure 14 and Table 5 show that the proposed method yields the best segmentation accuracy in most cases for both the WM and GM regions.

**Table 5:** Segmentation accuracy of WM and GM regions (in terms of Jaccard index × 100) using VLSBCS, LSACM and proposed methods.

Regions	VLSBCS	LSACM	Proposed
WM	87.32 ± 1.60	53.82 ± 1.01	**91.28** ± **0.33**
GM	66.04 ± 2.16	71.63 ± 1.53	**72.89** ± **0.88**

### Conclusions and future work

In this paper, a novel edge-based active contour method is proposed that uses a difference of Gaussians (DoG) function as an edge-indicator in its formulation. DoG function uses differences of two smooth images to extract edge information in an image, which acts as a balloon force in the energy functional to evolve the level set curve. In the proposed formulation, the internal energy term penalizes the deviation of the level set function from a signed distance function (SDF) and external energy term evolves the contour towards the boundaries of the objects.

The inclusion of DoG function in the energy formulation work as a global edge extractor and is able to segment all the regions in an image. Moreover, it is also able to properly segment images with intensity inhomogeneity. The results show that proposed method yields best segmentation results using both synthetic and real images as compared to the discussed state-of-the-art methods.

The proposed method segments all regions in an image globally; therefore, it cannot be used in the selective segmentation of a particular region in an image. In future, we would like to formulate a method to segment a selective region in an image, which can be a good help to segment particular regions in medical applications. The proposed method can properly segment intensity inhomogeneous images; however, it is unable to correct the bias of the inhomogeneous regions. In future, we would also like to formulate an edge-based active contours which can also correct the inhomogeneity of the regions.

## References

[1] Batenburg KJ, Sijbers J (2009). Adaptive thresholding of tomograms by projection distance minimization. Pattern Recognit..

[2] Barghout L, Sheynin J (2013). Real-world scene perception and perceptual organization: Lessons from computer vision. J. Vis..

[3] Guberman S, Maximov VV, Pashintsev A (2012). Gestalt and image understanding. Gestalt Theory.

[4] Ohlander R, Price K, Reddy DR (1978). Picture segmentation using a recursive region splitting method. Comput. Graph. Image Process..

[5] Kimmel R (2003). Fast Edge Integration. Geom. Level Set Methods in Imaging Vis. Graph..

[6] Nock R, Nielsen F (2004). Statistical region merging. IEEE Transactions on Pattern Analysis and Mach. Intell..

[7] Chen L, da Cheng H, Zhang J (1994). Fuzzy subfiber and its application to seismic lithology classification. Inf. Sc. – Appl..

[8] Kass M, Witkin A, Terzopoulos D (1988). Snakes: Active contour models. Int. J. Comput. Vis..

[9] Osher S, Sethian JA (1988). Fronts propagating with curvature-dependent speed: Algorithms based on Hamilton-Jacobi formulations. J. Comput. Phys..

[10] Caselles V, Kimmel R, Sapiro G (1997). Geodesic Active Contours. Int. J. Comput. Vis..

[11] Osher, S. & Paragios, N. *Geometric level set methods in imaging*, *vision*, *and graphics* (2003).

[12] Chan TF, Vese LA (2001). Active contours without edges. IEEE Transactions on Image Process..

[13] Mumford D, Shah J (1989). Optimal approximations by piecewise smooth functions and associated variational problems. Commun. Pure Appl. Math..

[14] Malladi R, Sethian J, Vemuri B (1995). Shape modeling with front propagation: a level set approach. IEEE Transactions on Pattern Analysis Mach. Intell..

[15] Li C, Xu C, Gui C, Fox M (2005). Level Set Evolution without Re-Initialization: A New Variational Formulation. 2005 IEEE Computer Society Conference on Computer Vision and Pattern Recognition (CVPR'05).

[16] Li C, Xu C, Gui C, Fox MD (2010). Distance regularized level set evolution and its application to image segmentation. IEEE Trans Image Process..

[17] Li C (2008). A variational level set approach to segmentation and bias correction of images with intensity inhomogeneity. Lecture Notes in Computer Science (including subseries Lecture Notes in Artificial Intelligence and Lecture Notes in Bioinformatics).

[18] Zhang K, Zhang L, Song H, Zhou W (2010). Active contours with selective local or global segmentation: A new formulation and level set method. Image Vis. Comput..

[19] Akram F, Kim J, Lee C, Choi KN (2015). Segmentation of regions of interest using active contours with SPF function. Comput. Math. Methods Medicine.

[20] Li, C., Kao, C. Y., Gore, J. C. & Ding, Z. Implicit active contours driven by local binary fitting energy. In *Proceedings of the IEEE Computer Society Conference on Computer Vision and Pattern Recognition* (2007). doi:10.1109/CVPR.2007.383014.

[21] Li C, Kao CY, Gore JC, Ding Z (2008). Minimization of region-scalable fitting energy for image segmentation. IEEE Transactions on Image Process..

[22] Zhang K, Song H, Zhang L (2010). Active contours driven by local image fitting energy. Pattern Recognit..

[23] Akram F, Kim J, Lim HU, Choi KN (2014). Segmentation of intensity inhomogeneous brain mr images using active contours. Comput. Math. Methods Medicine.

[24] Lankton S, Tannenbaum A (2008). Localizing region-based active contours. IEEE Transactions on Image Process..

[25] Li C (2011). A level set method for image segmentation in the presence of intensity inhomogeneities with application to MRI. IEEE Transactions on Image Process..

[26] Zhang K, Liu Q, Song H, Li X (2015). A variational approach to simultaneous image segmentation and bias correction. IEEE Transactions on Cybern..

[27] Zhang K, Zhang L, Lam K, Zhang D (2016). A level set approach to image segmentation with intensity inhomogeneity. IEEE Transaction on Cybern..

[28] Akram F, Garcia MA, Puig D (2017). Active contours driven by local and global fitted image models for image segmentation robust to intensity inhomogeneity. Plos One.

[29] Sethian, J. A. *Level set methods and fast marching methods* (1999). 10.1073/pnas.93.4.1591 PMC39986 11607632

[30] Caselles V, Catté F, Coll T, Dibos F (1993). A geometric model for active contours in image processing. Numer. Math..

[31] Evans, L. C. *Partial Differential Equations*, vol. 19 (1998).

[32] Jaccard P (1912). The distribution of the flora in the alphine zone. The New Phytol..

[33] Dice LR (1945). Measures of the Amount of Ecologic Association Between Species. Ecol..

[34] Matthews BW (1975). Comparison of the predicted and observed secondary structure of T4 phage lysozyme. BBA - Protein Struct..

[35] Huttenlocher DP, Klanderman GA, Rucklidge WJ (1993). Comparing Images Using the Hausdorff Distance. IEEE Transactions on Pattern Analysis and Mach. Intell..

[36] Aubert-Broche B, Griffin M, Pike GB, Evans AC, Collins DL (2006). Twenty new digital brain phantoms for creation of validation image data bases. IEEE Transactions on Med. Imaging.

[37] Aubert-Broche, B. *Brain web: Anatomical models of 20 normal brains* (2006). http://brainweb.bic.mni.mcgill.ca/brainweb/anatomic_normal_20.html [Accessed: April 24, 2015].

